# Presence and prevalence of single nucleotide sequence polymorphisms in *TP53* and *BRCA2* genes as of cervical and ovarian cancers in women using hormonal contraceptives in Abuja, Nigeria

**DOI:** 10.4102/jphia.v17i1.1453

**Published:** 2026-03-13

**Authors:** Omorinsola F. Odebiyi, Adedapo Kehinde, Mohammed S. Abdulsalami, Deborah M. Dibal

**Affiliations:** 1 Department of Biotechnology, Faculty of Sciences, Nigeria Defence Academy, Kaduna, Nigeria; 2 Department of Histopathology and Cytopathology, Faculty of Medical Laboratory Science, Achievers University, Owo, Nigeria

**Keywords:** cancer, SNPs, OVC, breast cancer, women’s health, TP53, BRCA

## Abstract

**Background:**

Cancer remains a major health burden in sub-Saharan Africa, characterised by high incidence and mortality rates as a result of late diagnosis, limited access to treatment and poor outcomes. Among gynaecological malignancies, cervical and ovarian cancers are of particular concern. Emerging evidence suggests that hormonal contraceptives may influence genetic susceptibility through single nucleotide polymorphisms (SNPs) in cancer-related genes such as *TP53* and *BRCA2*.

**Aim:**

This study investigated the prevalence of SNPs in the *TP53* and *BRCA2* genes and their association with cervical and ovarian cancer risk in a selected population.

**Setting:**

Civil Defence Medical Centre, Abuja, Nigeria.

**Methods:**

A total of 108 samples were analysed using polymerase chain reaction-restriction fragment length polymorphism (PCR-RFLP) to detect gene polymorphisms. Single nucleotide polymorphisms were confirmed at the nucleotide level using purified PCR products, and deoxyribonucleic acid (DNA) fragments were sequenced with a Genetic Analyzer 3130xl (Applied Biosystems). BioEdit and MEGA 6 software were used for genetic analysis.

**Results:**

Polymorphisms were detected in the *TP53* gene, but not in BRCA2. The TP53 variants were predominantly missense mutations, including G > A (40%) and G > C (60%) substitutions. Among 98 hormonal contraceptive users, 5 (5.1%) had TP53 SNPs. No BRCA2 SNPs were identified. Fisher’s exact test showed marginal statistical significance (*p* = 0.059).

**Conclusion:**

The findings underscore the relevance of TP53 screening in populations at risk of cervical cancer, while BRCA2 screening may be less applicable in this cohort. Larger studies across diverse populations are recommended.

**Contribution:**

However, further research is needed with larger sample sizes in other geographical regions and additional genetic markers to fully understand the genetic risk landscape for both cancers in hormonal contraceptive users within the population.

## Introduction

Cancer, a group of diseases characterised by the uncontrolled proliferation of abnormal cells, remains a leading cause of mortality worldwide, surpassing deaths caused by human immunodeficiency virus (HIV) and/or acquired immunodeficiency syndrome (AIDS), tuberculosis and malaria combined.^1^ The burden is particularly severe in sub-Saharan Africa, where cancer incidence and mortality rates continue to rise because of late-stage diagnosis, inadequate access to treatment and poor treatment outcomes.^2^ According to the 2018 report by the Federal Ministry of Health, cancer accounts for approximately 72 000 deaths annually, with about 102 000 new cases each year in Nigeria.^3^ Among gynaecological malignancies, cervical and ovarian cancers pose significant public health challenges because of their high prevalence and mortality rates.

Cervical cancer is the second most common cancer in women worldwide,^4^ with approximately 530 000 new cases annually, 85% of which occur in developing countries.^5^ Persistent infection with high-risk human papillomavirus (HPV), particularly HPV-16 and HPV-18, is the primary etiological factor, though other contributing factors include smoking, prolonged hormonal contraceptive use, genetic susceptibility and immunosuppression.^6,7^ Despite advancements in screening, late-stage diagnosis remains a significant concern in Nigeria because of limited access to healthcare services.^8^ Ovarian cancer, a malignancy originating from the ovary or fallopian tube epithelium, is often diagnosed at an advanced stage because of the absence of specific early symptoms, leading to a poor prognosis and high mortality rates.^9^ Recent evidence suggests that many ovarian cancers may originate in the distal fallopian tubes rather than the ovarian surface epithelium, highlighting the complexity of its aetiology.^10^ The development of ovarian cancer is influenced by genetic mutations, hormonal factors and inflammatory processes, with BRCA2 mutations significantly increasing susceptibility.

Hormonal contraceptives, widely used for birth control, contain synthetic steroid hormones that regulate the endocrine system. While they effectively prevent pregnancy, concerns have emerged regarding their potential role in cancer development. Research suggests that hormonal contraceptives may contribute to cervical and ovarian cancer risk by influencing genetic susceptibility, particularly through single nucleotide polymorphisms (SNPs) in cancer-related genes such as *TP53* and *BRCA2*.^11^ SNPs are genetic variations that can affect disease susceptibility, and mutations in BRCA2, a key deoxyribonucleic acid (DNA) repair gene, significantly increase ovarian cancer risk by impairing genomic stability. Similarly, cervical cancer has been linked to SNP variations in TP53, a critical tumour suppressor gene involved in apoptosis and cell cycle regulation.^12^ The influence of hormonal contraceptives on cancer susceptibility is thought to be mediated through hormonal regulation of gene expression, epigenetic modifications and interactions with HPV-related oncogenesis.^13^ Epidemiological studies indicate that oral contraceptives may alter cancer risk, with some findings suggesting an increased susceptibility to cervical cancer because of prolonged oestrogen exposure, while others report a reduced risk of ovarian cancer attributed to ovulation suppression; however, data on SNP variations in TP53 and BRCA2 among hormonal contraceptive users in Nigeria remain scarce, necessitating further investigation into these genetic markers. The study assessed SNPs in *TP53* and *BRCA2* genes among hormonal contraceptive users in Abuja, Nigeria, to better understand genetic predisposition to cervical and ovarian cancers. Given the rising incidence of gynaecological malignancies and the widespread use of hormonal contraceptives, investigating these genetic factors could provide valuable insights into cancer risk assessment, personalised contraceptive choices and public health interventions in Nigeria.

## Research methods and design

### Research design

This study adopted a case-control research design to investigate the prevalence and risk of SNPs in *TP53* and *BRCA2* genes in cervical and ovarian cancers in Nigerian women using hormonal contraceptives.

### Research setting and study area

This was a case-control study carried out in the family planning unit in the Civil Defence Medical Centre, Abuja. Abuja is both a Federal Capital Territory within the nation of Nigeria and a city within that territory which serves as the nation’s capital. It is slightly west of the centre of the country. Abuja lies at 1180 feet (360 m) above sea level; the latitude of Abuja is 9.072264 and the longitude is 7.491302, and its area covers 2824 square miles (7315 square km). The Federal Capital Territory falls within the Savannah Zone vegetation of the West African sub-region.^14^

### Study population

The study population consisted of women between the ages of 18 years and 60 years on hormonal contraceptives who will be attending the family planning unit in the Civil Defence Medical Centre. They were recruited from the Nigeria Security and Civil Defence Corps Medical Centre, Abuja. The control groups were recruited from the same hospitals and were non-contraceptive (hormonal) users at any point in time in their lives. Basic demographic biodata was collected via a questionnaire on the type of contraceptive, administration route and usage duration.

### Sampling technique and sample size determination

These sample selections were carried out through a random sampling technique as described by Naing^15^ and Usiobeigbe.^16^ A total of 108 samples were obtained for the study, with 98 individuals categorised as hormonal contraceptive users and 10 as non-users that served as a control.

### Sample and data collection

Five millilitres of venous blood were collected aseptically without stasis from the prominent vein of the cubital fossa and dispensed into potassium EDTA (K_3_EDTA) bottles. The whole blood was stored at −20 °C until analysed. Participant data were collected using a structured questionnaire.

### Inclusion criteria

Inclusion criteria include subjects between the ages of 18 years and 60 years that were on hormonal contraceptives of any type and had not been diagnosed with breast cancer or ovarian cancer. Control subjects were women who had never taken any form of contraceptive – combined, single or emergency – and also free of, either and both, breast and/or ovarian cancer.

### Exclusion criteria

Subjects that have reached menopause, are below the age of 18 years or above 60 years or are currently undergoing treatment for metabolic disorders were excluded, and those who had these diseases before taking hormonal contraception were also excluded, and those who refused to give consent were excluded from this study.

### Molecular detection of single nucleotide polymorphisms in TP53 and BRCA2 using polymerase chain reaction–restriction fragment length polymorphism

To investigate SNPs in the *TP53* and *BRCA2* genes, we employed the polymerase chain reaction–restriction fragment length polymorphism (PCR-RFLP) technique as previously described by Chang et al.^17^ and Tarach,^18^ and with slight modifications by Damin.^19^ This method enables rapid and cost-effective genotyping of known point mutations by leveraging sequence-specific restriction enzyme digestion of PCR-amplified DNA, and the robust PCR-RFLP protocol enabled precise discrimination of allelic variants in TP53 and BRCA2, making it a reliable approach for genotyping SNPs associated with cancer susceptibility in small- to medium-scale molecular epidemiological studies.

Genomic DNA was extracted from peripheral whole blood samples using the QIAamp DNA Mini Kit (Qiagen, Germany) following the manufacturer’s instructions. Briefly, 200 µL of blood was lysed, mixed with ethanol and passed through a spin column for purification, followed by washes and elution with Tris-EDTA elution buffer (AE). Purified genomic DNA was stored at −20 °C until further use. DNA quality was assessed using a NanoDrop spectrophotometer (Thermo Fisher Scientific, Waltham, Massachusetts, United States). Samples were considered suitable for downstream analysis based on the following predefined benchmarks: an A260/280 absorbance ratio between 1.8 and 2.0, indicating acceptable protein-free DNA purity, and a minimum DNA concentration of 20 ng/µL. Only samples meeting these criteria were selected for PCR analysis.

Polymerase chain reaction amplification was carried out in a total reaction volume of 25 µL, comprising 12.5 µL of 2× PCR Master Mix (Promega, United States), 0.2 µM of each forward and reverse primer specific to SNP loci in TP53 and BRCA2 and 50 100 ng of genomic DNA as a template (corresponding to a minimum template concentration of 2 ng/µL per reaction). Nuclease-free water was added to complete the reaction volume. Negative controls containing no template DNA were included in each PCR run to monitor contamination. Thermocycling consisted of initial denaturation at 94° C, followed by 40 cycles of denaturation, annealing at 48 °C and extension and finalised with an elongation step at 72 °C. Following amplification, PCR products were digested with appropriate restriction enzymes such as EcoRI and MspI in 20 µL reactions at 37 °C, following the manufacturer’s guidelines, to detect polymorphisms affecting restriction sites.

The restriction fragments generated were analysed using 2% (weight per volume [w/v]) agarose gel electrophoresis pre-stained with ethidium bromide. Deoxyribonucleic acid fragments, being negatively charged, migrated towards the positive electrode when an electric field was applied, with smaller fragments moving faster than larger ones, resulting in separation based on size. Fragmented samples were loaded into the gel chamber, and after electrophoresis, the gels were treated with ethidium bromide (0.5 µg/mL for 10 min) to enhance DNA band visibility. Bands were visualised using an ultraviolet (UV) transilluminator (Dark Reader X), and photographic records were taken. Fragment sizes were compared to a molecular weight marker to determine SNP genotypes based on digestion patterns observed.

### Single nucleotide polymorphisms confirmation at the nucleotide level

Subsequently, the purified samples from the PCR technique were used for amplification of the DNA fragments, which were sequenced using a Genetic Analyzer 3130xl sequencer from Applied Biosystems using the manufacturers’ manual; Bio-Edit software (version 7.2) and Mega 6 were used for all genetic analysis, as described by Njoko^20^ and modified by Olukayode.^2^

### Statistical analysis

Data were analysed using the Chi-square test of independence, Fisher’s exact test and regression analysis. Quantitative and qualitative variables were summarised using frequencies, tables, charts and pictorial diagrams. Appropriate statistical tools within Microsoft Excel were used to determine the significance of the result; the level of significance was set at *p* ≤ 0.05.

### Ethical considerations

Ethical clearance to conduct this study was obtained from the Nigeria Security and Civil Defence Corps, National headquarters, Abuja (reference number: NSCDC/NHQ/HMSU/023/04). Participants’ consent was obtained along with a consent form.

## Results

The study involved a total of 108 participants from women attending the family planning unit in Civil Defence Medical Centre, Abuja, with 98 individuals categorised as hormonal contraceptive users and 10 as non-users (controls). Molecular analysis was conducted using PCR-RFLP, followed by sequence alignment through National Center for Biotechnology Information’s (NCBI) Basic Local Alignment Search Tool (BLAST) to identify SNPs, while statistical tests were employed to evaluate the distribution and significance of observed mutations.

### Biodata distribution of the participants during the research study

The total number of samples collected was 108; this number encompassed different age ranges, ethnic groups, religions, marital status, education backgrounds, employment and duration of contraceptive usage and various types of hormonal contraceptives, as shown in Table 1. The data from the questionnaire shows that the age bracket of 25–31 years old showed the highest frequency (*n* = 28) and percentage (26%), and ages 53–60 years showed the lowest frequency (*n* = 6) and percentage (6%). The Hausa ethnic group had the highest frequency of 60 (55.5%), and the Christian religion had a frequency of 66 (61%). A high number of the participants were married, with a frequency of 68 (63%), and most have received a tertiary level of education, with a frequency of 44 (41%); however, the majority had a semi-skilled form of employment, with a frequency of 35 (32%). Also, the majority of the participants had been on one form of hormonal contraceptives for at least 5 years and above, with a frequency of 59 (55%), and having injectable types of hormonal contraceptives was the highest form of contraceptives used, with a frequency of 40 (37%).

**TABLE 1:** Amplification of *TP53* and *BRCA2* genes using polymerase chain reaction.

Type	Sequence
TP53 Forward	CCCACCTCTTACCGATTTCTTC
TP53 Reverse	CCGTCCCAGTAGATTACCACTA
BRCA2 Forward	CGCTGCAACAAAGCAGATTTA
BRCA2 Reverse	CTCCCACATACCACTGACTTATC

Source: Adapted from Chansaenroj J, Theamboonlers A, Junyangdikul P, et al. Polymorphisms in TP53 (rs1042522), p16 (rs11515 and rs3088440) and NQO1 (rs1800566) genes in Thai cervical cancer patients with HPV 16 infection. Asian Pac J Cancer Prev. 2013;14(1):341–346. https://doi.org/10.7314/APJCP.2013.14.1.341; Tsibulak I, Wieser V, Degasper C, et al. BRCA1 and BRCA2 mRNA-expression prove to be of clinical impact in ovarian cancer. Br J Cancer. 2018;119:683–692. https://doi.org/10.1038/s41416-018-0217-4

**TABLE 2:** Biodata of participants during the research study.

Variables	Group	Frequency (*n*)	%
Age (years)	18–24	19	18
	25–31	28	26
	32–38	25	23
	39–45	14	13
	46–52	16	15
	53–60	6	6
Ethnic group	Igbo	23	21
	Yoruba	21	19
	Hausa	60	56
	Others	4	4
Religion	Christian	66	61
	Islam	40	37
	Traditional	2	2
Marital status	Single	13	12
	Married	68	63
	Divorced or separated	19	18
	Widow	8	7
Education	No formal	21	19
	Primary	11	10
	Secondary	32	30
	Tertiary	44	41
Employment	Unemployed	23	21
	Unskilled	27	25
	Semi-skilled	35	32
	Skilled	23	21
Duration of contraceptives	Never	10	9
	1–2 years	24	22
	3–5 years	37	34
	5 years above	59	55
Type of hormonal contraceptives used	Implants	30	27
	Injectables	40	37
	Intra-uterine devices	16	15
	Oral	12	11
	Non-user (No hormonal contraceptives)	10	9

### Molecular findings

PCR-RFLP analysis followed by gel electrophoresis revealed the presence of SNPs in the *TP53* gene in a subset of the test group. In contrast, no *BRCA2* gene polymorphisms were detected in the participant samples. Sequence analysis using NCBI BLAST confirmed both wild-type and variant isoforms of the *TP53* gene. Two types of mutations were observed in the *TP53* gene: G > A transition: 2 cases (40%) and G > C transversion: 3 cases (60%). These mutations resulted in missense changes, potentially altering protein function.

Polymorphisms on the *TP53* gene for cervical cancer were observed in some samples, which was indicated by the restriction enzyme not being able to cleave the right sequence of the gene and showing variants of shortening of the bands on the electrophoresis gel. The nucleotide sequence was blasted on the NCBI website, which confirms the presence of isoforms of the *TP53* gene nucleotide sequences. It was observed that missense mutations were caused by changes in the nucleotide sequences.

However, polymorphisms of the *BRCA2* gene for ovarian cancer were not seen in samples from participants from the present study. This was indicated by the restriction enzyme not being able to cleave the right sequence of the gene to show a shortened band on the electrophoresis gel, and when blasted on the NCBI website, it returned the normal nucleotide sequence for the *BRCA2* gene, which means that polymorphism did not occur.

### Single nucleotide polymorphisms prevalence in TP53

Out of the 98 hormonal contraceptive users, 5 were found to have TP53 SNPs, giving a prevalence rate of approximately 5.1%. BRCA2 SNPs were absent across all participants.

### Association between TP53 mutation and contraceptive type

A Chi-square test of independence was performed to determine if the type of hormonal contraceptive used had any significant association with the presence of TP53 SNPs. The contingency table (Table 1)^21, 22^ included data from injectables, implants, intrauterine devices (IUD), oral contraceptives and non-users (χ^2^ = 1.08, degree of freedom [*df*] = 4, *p* = 0.897). The results did not show statistical significance (*p* > 0.05), indicating no significant association between contraceptive type and TP53 SNP prevalence.

### Comparison of TP53 and BRCA2 single nucleotide polymorphisms prevalence

To compare the prevalence of SNPs between the *TP53* and *BRCA2* genes, Fisher’s exact test was applied (*p* = 0.059). It showed a marginally close statistical significance (*p* > 0.05).

## Discussion

Because of the large cultural belief among Nigerians against abortion coupled with it being illegal in the country, unless medically recommended by the doctor to save the mother’s life, many turn to hormonal contraceptives as a preventive measure to not have children rather than actually aborting pregnancies in unsafe environments, and this has caused a leaping rise in the number of hormonal contraceptive users in the country.^23^

The use of injectable contraceptives, which reduces the need for daily consumption of contraceptives (e.g. combinational pills), has made it one of the widely used methods of contraception; its growing use can also be attributed to its desirable characteristics, cost-efficient nature, ease of use, reversibility and long-acting effect. Some women on injectable contraceptives have complained that it has reduced the frequency of their periods, which other new users have taken as a side advantage.

However, hormonal contraception, with its advantages in reducing population size by reducing the number of newborns, which in turn leads to economic growth, and also in reducing unsafe abortions, is found to be associated with increased susceptibility to and development of cervical and ovarian cancers. The National Cancer Institute (NCI)^24^ reported that hormonal contraceptive use is a cofactor in the formation and development of cervical and ovarian cancers and also in making the user predisposed to having the disease conditions in the first place. This study aimed to evaluate the prevalence of SNPs in the *TP53* and *BRCA2* genes and their potential association with cervical and ovarian cancers in a selected population; through PCR-RFLP and BLAST analysis as described in Table 3 and Table 4, polymorphisms were only observed in the TP53 gene and the frequency is shown in Table 5.

**TABLE 3:** NCBI BLAST showing the identity of the fragment amplified with TP53-specific primers.

Sample ID	Description	Query cover (%)	*E* value	% identity
TP1	*Homo sapiens* TP53 isoform	100	0	99.64
TP2	*Homo sapiens* TP53 isoform	100	0	99.64
TP3	*Homo sapiens* TP53 isoform	100	0	99.82
TP4	*Homo sapiens* TP53 germline	100	0	100.00
TP5	*Homo sapiens* TP53	100	0	99.82
TP6	*Homo sapiens* TP53 variant Isoform	100	0	100.00
TP7	*Homo sapiens* TP53 isoform	100	0	100.00
TP8	*Homo sapiens* TP53	100	0	99.64
TP9	*Homo sapiens* TP53	100	0	99.82
TP10	*Homo sapiens* TP53	100	0	99.82

NCBI, National Center for Biotechnology; BLAST, Information’s Basic Local Alignment Search Tool.

**TABLE 4:** NCBI BLAST showing the identity of the fragment amplified with BRCA2-specific primers.

Sample ID	Description	Query cover (%)	*E* value	% identity
BRC1	*Homo sapiens* BRCA2 gene	100	0	100.00
BRC2	*Homo sapiens* BRCA2 gene	100	0	99.84
BRC3	*Homo sapiens* BRCA2 gene	100	0	100.82
BRC4	*Homo sapiens* BRCA2 gene	100	0	99.79
BRC5	*Homo sapiens* BRCA2 gene	100	0	99.82
BRC6	*Homo sapiens* BRCA2 gene	100	0	100.00
BRC7	*Homo sapiens* BRCA2 gene	100	0	99.89
BRC8	*Homo sapiens* BRCA2 gene	100	0	99.64
BRC9	*Homo sapiens* BRCA2 gene	100	0	99.82
BRC10	*Homo sapiens* BRCA2 gene	100	0	100.00

NCBI, National Center for Biotechnology; BLAST, Information’s Basic Local Alignment Search Tool.

**TABLE 5:** Single nucleotide polymorphisms observed in TP53 gene among participants.

Description	Number of occurrence (frequency)	Location	Gene	Mutation description	Mutation type	Percentage of mutation signature
G > A	2	24(7:3)	*TP53*	Transition	Missense (Glycine to aspartate)	40.00
G > C	3	64(9:1)	*TP53*	Transversion	Missense (Arginine to serine)	60.00

### Biodata of participants

The biodata distribution of the participants in this research study, as shown in Table 2 provides valuable insight into the demographic characteristics of the participants of the sample population. A total of 108 women participated in the study, the majority of whom were users of hormonal contraceptives. The data were analysed across various parameters, including age, ethnicity, religion, marital status, educational background, employment status and duration of contraceptive use. These variables play significant roles in influencing health outcomes, including cancer risk factors, as they reflect underlying socioeconomic and cultural factors that may impact access to healthcare, lifestyle choices and genetic predispositions.

### *TP53* gene mutations and cervical cancer risk

**FIGURE 1:**
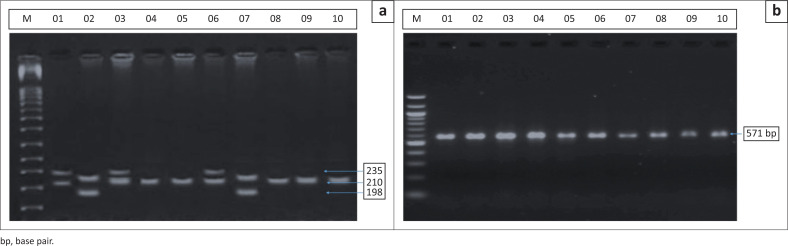
Agarose gel demonstrating positive polymerase chain reaction genotyping of genomic deoxyribonucleic acid samples for detection of TP53 genotypes.

**FIGURE 2:**
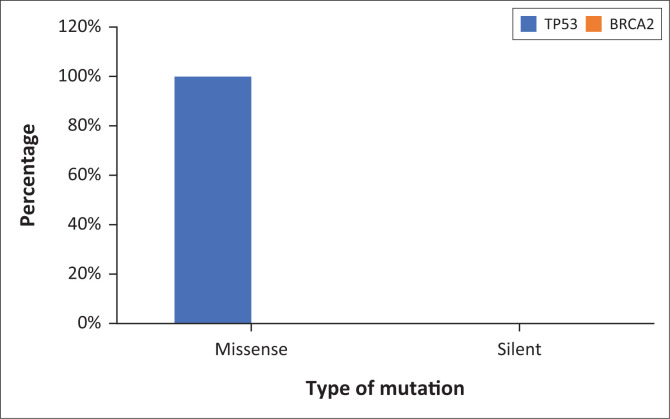
Functional mutation types observed in TP53 and BRCA2 in participants: Statistical analysis.

In this research study, SNPs were observed in the *TP53* gene in 5.1% of the samples, as shown in Figure 1 this is similar to reports in the established literature linking TP53 mutations to an increased risk of cervical cancer, similar to a cohort study by Mørch et al.^25^ The most common polymorphisms detected were G > A (40%) and G > C (60%), both of which were missense mutations as shown in Figure 2. These mutations lead to amino acid substitutions that could disrupt the tumour-suppressor functions of the TP53 protein. Specifically, the G > A transition results in a glycine to aspartate substitution, while the G > C transversion leads to an arginine to serine substitution. Both mutations are known to alter the structure and function of TP53, impairing its role in DNA repair, apoptosis and cell cycle regulation.

This is consistent with several studies, such as one by Petitjean et al.^26^, which highlights that TP53 mutations are among the most frequent alterations in cervical cancer patients and are associated with more aggressive tumour phenotypes and poorer prognoses. Studies by Mehta & Singh^27^ also indicate that TP53 mutations play a key role in the development of cervical and other cancers, further supporting the findings of this research. Additionally, Hu & Wu^28^ emphasise that TP53 mutations are often linked to cancer progression and poor treatment outcomes, making early detection of these polymorphisms vital for improved patient prognosis. Therefore, the presence of these mutations in the sampled population suggests a heightened risk of developing cervical cancer, especially among individuals with these specific polymorphisms.

### Absence of BRCA2 polymorphisms in ovarian cancer

In contrast, no polymorphisms were detected in the *BRCA2* gene in the samples analysed, as shown in Figure 1. This result is somewhat unexpected, as BRCA2 mutations are strongly associated with an increased risk of ovarian cancer, as reported by Rookus et al.^29^ and Phillips et al.^30^, who found a slightly increased risk of breast cancer for BRCA/12 mutation carriers who ever used oral contraceptives. The absence of BRCA2 mutations in this study could be attributed to several factors. Firstly, the sample size might not have been large enough to detect rare polymorphisms in BRCA2. Secondly, other genetic factors or environmental influences, such as HPV infection, might play a more prominent role in the aetiology of ovarian cancer in this population.

Other studies, such as those by Kuchenbaecker et al.^31^, emphasise that not all ovarian cancer cases are linked to BRCA mutations, and some patients may develop ovarian cancer through alternative genetic pathways. Furthermore, Collins et al.^32^ discuss that while BRCA mutations are significant risk factors, ovarian cancer can also develop independently of *BRCA* gene mutations, suggesting other genetic and environmental interactions might be at play. Therefore, while BRCA2 mutations are a well-established risk factor, their absence in this study does not necessarily negate the genetic predisposition to ovarian cancer in this population.

### Implications of findings

The findings from this study have significant implications for understanding the genetic risk factors associated with cervical and ovarian cancer in this population. The presence of TP53 polymorphisms in women on hormonal contraceptives indicates a potential genetic predisposition to cervical cancer; this reinforces the need for early genetic screening and preventive strategies among high-risk individuals. Screening for TP53 mutations could aid in identifying women at higher risk and facilitate timely intervention, which could improve outcomes.

On the other hand, the absence of BRCA2 polymorphisms suggests that BRCA screening may not be as critical for ovarian cancer risk assessment in this particular population. However, considering the small sample size and the localised nature of the study, further research with larger cohorts and additional genetic markers is necessary to draw more definitive conclusions.

Compared to global data, the results of this study are in line with reports that TP53 mutations are prevalent in cervical cancer cases. For example, a study by Wang et al.^33^ found similar missense mutations in TP53 to be highly predictive of cervical intraepithelial neoplasia progressing to invasive cervical cancer. Conversely, the lack of BRCA2 mutations contrasts with studies conducted in other populations, where BRCA2 mutations were more frequently observed. This suggests a potential geographical or ethnic variation in the prevalence of these mutations, highlighting the importance of population-specific cancer genetics research.

### Limitations

The category of women attending the clinic is mostly on one form of contraceptives, giving a difficulty in getting a larger number of women in the non-contraceptives category, resulting in a small number of control samples.

The objective of the research was focused on only two categories (non-contraceptive users versus hormonal contraceptive users), so the results of the research were in consideration of how hormonal contraceptives affect SNPs of TP53 and BRCA2.

The genotype of the controls was not reported, because they showed a negative SNP.

### Recommendations

There is a need for enhancing nationwide awareness of the effect of hormonal contraceptive usage, conducting further studies to determine the specific type of hormonal contraceptive that primarily predisposes patients to cancers, conducting further studies on other genes with specific restriction enzymes, conducting multi-site studies with larger population sizes to guide decision-making regarding contraceptive use and disuse, and routinely screening women on contraceptives to monitor their effects on the human body. Future studies should aim to include larger sample sizes and explore additional genetic markers, as well as consider the interaction between genetic, environmental and lifestyle factors in the development of cervical and ovarian cancers.

### Future research considerations

- Future research should consider stratifying the SNP findings based on the specific hormonal contraceptive group among the participants within a larger population.
- The genotype of the control samples should be reported and juxtaposed with the SNP findings to help collate and compare the direct effect of contraceptive usage.

## Conclusion

In summary, this study identified significant polymorphisms in the *TP53* gene, suggesting a genetic predisposition to cervical cancer among the participants using one form of hormonal contraceptives. However, no BRCA2 polymorphisms were detected, indicating that BRCA-related ovarian cancer risk might be lower in this population. These findings underscore the importance of genetic screening for TP53 mutations in cervical cancer prevention.
